# Effects of exercise on anxiety and psychiatric comorbidities in children with autism spectrum disorder: a systematic review and meta-analysis

**DOI:** 10.3389/fpsyt.2026.1761833

**Published:** 2026-02-20

**Authors:** Liqiang Zhang, Wenkun Song, Huina Gao, Xingying Li

**Affiliations:** 1 School of Physical Education, Xizang Minzu University, Xianyang, Shaanxi, China; 2 Department of Physical Education, Shaanxi University of International Trade and Commerce, Xianyang, Shaanxi, China; 3 College of Physical Education and Health, East China Normal University, Shanghai, China

**Keywords:** aerobic training, anxiety, autism spectrum disorder, exercise, meta-analysis

## Abstract

**Background:**

Anxiety disorders affect nearly 40%–50% of children with autism spectrum disorder (ASD). While exercise benefits motor skills, its efficacy in modulating affective symptoms remains under-synthesized. This meta-analysis evaluated the effects of exercise on anxiety and co-occurring symptoms in pediatric ASD.

**Methods:**

We analyzed data from 12 randomized controlled trials (RCTs) involving 482 participants. A three-level random effects model was utilized to account for effect size dependencies. Subgroup analyses examined the exercise modality and intensity.

**Results:**

Exercise significantly reduced comorbid anxiety (Hedges’ *g* = −0.68, *p* < 0.001). Notably, aerobic exercise demonstrated the most substantial anxiolytic effect (*g* = −1.18), outperforming other modalities. Improvements were also observed in the core ASD symptoms (*g* = −0.56) and attention-deficit/hyperactivity disorder (ADHD)-related attention deficits (*g* = −0.48). The effects on sleep were inconclusive due to heterogeneity.

**Conclusions:**

Exercise, particularly aerobic interventions, serves as a potent non-pharmacological strategy for the management of anxiety and affective dysregulation in children with ASD. These findings support the integration of aerobic exercise into clinical treatment plans to improve emotional wellbeing.

**Systematic review registration:**

https://www.crd.york.ac.uk/prospero/, identifier CRD420251157119.

## Graphical Abstract

**Figure:**
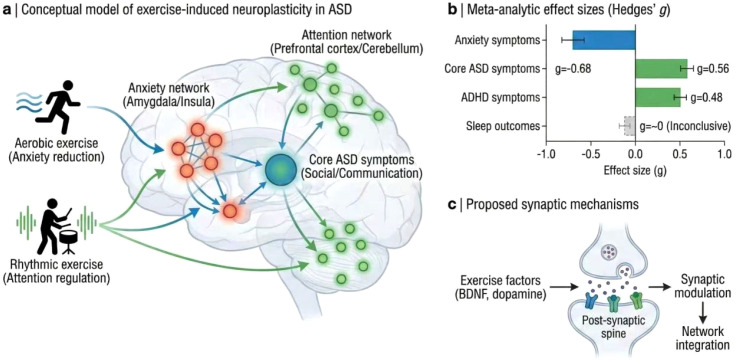
Mechanistic diagram or graphical abstract illustrating how exercise interventions promote improvements in the core and co-occurring symptoms in children with autism spectrum disorder (ASD).

## 1 Introduction

Autism spectrum disorder (ASD) is characterized by social communication deficits and restricted, repetitive behaviors (1). Beyond these core features, psychiatric comorbidities are highly prevalent. Epidemiological data indicate that 40%–50% of individuals with ASD meet the clinical criteria for anxiety disorders, while comorbidity with attention-deficit/hyperactivity disorder (ADHD) ranges from 30% to 80% (2, 3). In addition, frequent sleep disturbances are associated with the exacerbation of repetitive behaviors and emotional problems. These co-occurring conditions exacerbate the core ASD symptoms and are associated with poorer prognosis and a reduced quality of life (4).

Clinically, anxiety disorders in school-aged children with ASD are often associated with social interaction difficulties, sensory sensitivity, and reactivity to unpredictable environments (5). Similarly, co-occurring ADHD symptoms frequently overlap with the core ASD features, manifesting as inattention, hyperactivity, and impulsivity (6). Sleep problems—including difficulties in initiating sleep, frequent nighttime awakenings, and irregular sleep–wake cycles—are also prevalent and may be linked to dysregulated melatonin secretion and atypical sensory processing (7).

Recent research has demonstrated growing interest in exercise-based interventions as non-pharmacological approaches for addressing the core symptoms of ASD and its common comorbidities (8). Accumulating evidence indicates that physical activity yields benefits across multiple functional domains (9), including enhancements in motor skills (10) and reductions in the core ASD symptoms (11). Specifically, interventions have been shown to reduce repetitive behaviors (12), improve social engagement and communication (13, 14), and enhance executive function (15). Beyond the core symptoms, exercise is recognized as an effective strategy for regulating anxiety and ADHD symptoms (16, 17), potentially through physiological mechanisms such as the promotion of neurotransmitter release (18). Consequently, preliminary evidence supports physical activity as a viable strategy to mitigating anxiety, sleep disturbances, and the ADHD-related deficits in children with ASD (19, 20).

Although evidence supports the benefits of exercise for ASD (21), a key limitation in the literature is that many of the studies approach ASD as a homogeneous condition, overlooking the heterogeneity arising from common comorbidities (22). Children with co-occurring anxiety, sleep disorders, or ADHD often differ substantially in terms of their neurobiological profiles and intervention needs (23). While interventions often target specific symptoms—such as the energy expenditure for ADHD (24) or relaxation for anxiety (25)—direct quantitative comparisons of the effects of exercise across these subgroups remain scarce. Majority of the existing meta-analyses have evaluated ASD or ADHD in isolation, failing to address concurrent presentations. This gap limits the development of precision exercise prescriptions (26). To our knowledge, no systematic review has yet evaluated the combined and comparative effects of exercise on co-occurring anxiety, sleep disorders, and ADHD in this population (27, 28).

Consequently, this systematic review and meta-analysis aimed to: 1) quantify the overall effect size of exercise interventions on the core ASD symptoms; 2) evaluate their effects on comorbid psychiatric conditions—specifically anxiety, sleep disturbances, and ADHD symptoms—via subgroup analyses; and 3) identify potential moderators of intervention efficacy (e.g., exercise modality, intensity, and duration) to generate evidence-based recommendations for clinical practice.

## 2 Methods

### 2.1 Research methodology

This systematic review and meta-analysis is pre-registered in the International Prospective Systematic Reviews Register (PROSPERO) (registration no. CRD420251157119).

### 2.2 Search strategy and selection criteria

We conducted a systematic search across the following electronic databases in both English and Chinese: PubMed, the Cochrane Library, Embase, Web of Science (WoS), PsycINFO, Science Direct, ProQuest, Scopus, and the China National Knowledge Infrastructure (CNKI). The search period spanned from the inception of each database up to September 1, 2025, in order to incorporate the most recent relevant literature. Our search strategy incorporated both controlled vocabulary (e.g., MeSH terms) and free-text keywords, with syntax adaption customized for each database.

The following were the search terms used:

*Population-related search terms*: ASD; autistic disorder; Asperger syndrome; pervasive developmental disorder; autism; ADHD; anxiety; sleep disturbance; insomnia; children; adolescents; and pediatric populations.
*Intervention-related search terms*: exercise; physical activity; physical training; sport; motor activity; motor skill training; yoga; swimming; aerobic exercise; resistance training; and exergaming.
*Study design search terms*: “randomized controlled trial”; “controlled clinical trial”; “randomized”; “placebo”; and “clinical trial”.

Boolean search strategies employ the OR operator to combine synonymous terms within a given concept and the AND operator to intersect different concepts. The following strategy was used:

(((Autism Spectrum Disorder [Mesh]) OR Autistic Disorder autist OR ASD OR Asperger Syndrome [Mesh]) AND ((attention deficit disorder with Hyperactivity [Mesh]) OR ADHD OR anxiety OR Sleep Wake Disorders [Mesh] OR insomnia) AND ((Exercise”[Mesh]) OR Motor Activity [Mesh] OR Physical Activity OR sport OR yoga OR swimming OR exergame) AND ((Randomized Controlled Trial OR Controlled Clinical Trial OR randomized OR placebo OR randomly) AND (child OR adolescent OR pediatric))).

In addition, the reference lists of relevant studies were manually searched to identify any potentially omitted research not captured by the electronic database searches. Field experts were also consulted to obtain information regarding unpublished studies, thereby mitigating the potential impact of publication bias.

### 2.3 Inclusion and exclusion criteria

The inclusion and exclusion criteria for the literature search were established based on the PICO(S) (Patient/Population, Intervention, Comparison, Outcomes, and Study) framework (**Table 1**).

**Table 1:** Inclusion criteria based on the PICO(S) (patient/population, intervention, comparison, outcomes, and study) framework.

PICO(S)	Intervention
Research population (P)	1) Children under the age of 18 2) Diagnosed with ASD by a professional in accordance with internationally recognized diagnostic criteria (such as the Diagnostic and Statistical Manual of Mental Disorders, DSM-IV/5 or the International Classification of Diseases, ICD-10/11) (29) 3) The study explicitly reported that participants had comorbid anxiety, sleep disorders, or ADHD symptoms, which were assessed using standardized scales or clinical diagnoses.
Intervention (I)	1) Any form of structured exercise intervention, including, but not limited to, aerobic exercise, resistance training, flexibility training, yoga, martial arts, dance, team sports, and motor skills training 2) The intervention must be systematic, with clearly defined frequency, intensity, single-session duration, and total cycle. The total intervention duration must be at least 4 weeks.
Comparison (C)	No-intervention placebo group, waiting list control group, treatment-as-usual group, or non-exercise activity control group
Outcomes (O)	1) The study must report at least one quantitative outcome measure related to the core ASD symptoms or comorbid symptoms. 2) The study must report quantitative data comparing pre- and post-intervention or between groups, from which these statistical measures can be calculated.
Study design (S)	1) Randomized controlled trials (RCTs) 2) Quasi-experimental studies

*ASD*, autism spectrum disorder; *ADHD*, attention-deficit/hyperactivity disorder.

The exclusion criteria were as follows:

1. Non-randomized controlled trials (RCTs), such as case reports, case series analyses, and review articles, among others;
2. Studies conducted on adults or animals;
3. Studies involving non-exercise interventions or those in which exercise is part of a multimodal intervention and its effect cannot be disentangled from other components;
4. Studies with unavailable full-text or insufficient data for extraction and analysis; and
5. Non-English or non-Chinese literature.

### 2.4 Literature screening and data extraction

Two independent reviewers performed the literature screening. They formulated the search strategy, managed the references using EndNote X21, and applied predefined inclusion criteria to identify relevant studies. Discrepancies between reviewers were minimal (<1%) and were resolved through discussion until consensus was achieved. In the subsequent stage, full-text articles were assessed by a third reviewer, who, in consultation with the primary reviewer, adjudicated all exclusion decisions. Consensus was reached for each excluded study.

Two reviewers independently extracted the study data using a pre-piloted data extraction form. Discrepancies were resolved through discussion with a third reviewer until consensus was reached. The extracted data included: authors and year of publication; country; sample size (with breakdown into experimental and control groups); mean age and standard deviation; sex distribution (male-to-female ratio); intervention modality; baseline frequency and session duration; intervention duration; outcome measures and assessment instruments; and other reported outcomes (**Supplementary material 2.4**).

### 2.5 Statistical analysis

Statistical analyses were performed in R (version 4.5.1) and Review Manager 5.4. To address the presence of multiple non-independent effect sizes reported by many of the included studies, a three-level (multilevel) meta-analytic model was fitted. To account for the dependency of effect sizes resulting from multiple outcomes reported within the same study, a three-level random effects meta-analytic model was employed. In this hierarchical model, sampling variance was treated as level 1, within-study variance (variation of effect sizes within the same study) as level 2, and between-study variance as level 3. This approach is superior to the standard random effects models for handling nested data structures as it prevents the inflation of type I error rates associated with treating dependent effect sizes as independent (30). Effect sizes were calculated as Hedges’ *g*, incorporating small-sample corrections and standardization in accordance with established methodological guidelines. To ensure consistent interpretation across all outcome measures, the effect directions were aligned by reversing the sign of the effect sizes for the inversely scored metrics, such that a negative *g* uniformly indicates symptom reduction. To ensure clarity in the interpretation of the effect directions, no sign reversal was applied for identical outcome measures; a negative Hedges’ *g* thus reflects a reduction in symptom severity in favor of the exercise group. All analyses were conducted in R (version 4.5.1) utilizing specialized packages for meta-analysis and data visualization, including metafor, ggplot2, ggridges, viridis, dplyr, tidyr, patchwork, RColorBrewer, ggrepel, and ggnewscale (31).

It should be noted that, in this meta-analysis, the following statistical indicators were used: *g* (Hedges’ *g*), which represents the standardized mean difference effect size; 95%CI indicates the 95% confidence interval around the point estimate; *p* indicates the statistical significance level; *I*^2^ quantifies the proportion of total variability due to between-study heterogeneity; *Q* corresponds to Cochran’s heterogeneity statistic; QM refers to the test statistic for moderators in the meta-regression; and beta (*β*) represents the regression coefficient in the meta-regression analyses. Together, these metrics provide the statistical estimation of the effect sizes, the heterogeneity assessment, and the moderator analysis.

### 2.6 Risk of bias and quality of evidence assessment

The Cochrane Risk of Bias 2.0 tool (RoB2) was used to evaluate the study-level risk of bias across five domains: 1) bias arising from the randomization process; 2) bias due to deviations from the intended interventions (performance bias); 3) bias due to missing outcome data (attrition); 4) bias in the measurement of the outcome (detection bias); and 5) bias in the selection of the reported results (reporting bias) (32). Each domain in RoB2 was classified as “low risk,” “some concerns,” or “high risk.” For an overall study-level evaluation, we assigned one point per domain judged as “low risk” and categorized the overall quality of each study into three tiers: high (≥5 points), moderate (3–4 points), and low (≤2 points). We acknowledge that this numerical scoring system is an adapted heuristic used solely for descriptive summary purposes in this review and is not part of the standard Cochrane RoB 2.0 methodology. Two reviewers independently evaluated the RoB2 domains for all included studies. Disagreements were resolved by discussion, with a third reviewer adjudicating unresolved discrepancies. We used the GRADE (Grading of Recommendations, Assessment, Development, and Evaluation) framework to assess the certainty of evidence across five domains: risk of bias (study limitations), inconsistency, indirectness, imprecision, and publication bias (33). Each domain was evaluated for potential downgrading based on the level of concern: no serious concern (no downgrade), serious concern (downgrade by one level), or very serious concern (downgrade by two levels). The overall certainty of evidence was then categorized in accordance with the GRADE criteria as high, moderate, low, or very low. The risk of bias assessments and the GRADE ratings are presented alongside forest plots.

### 2.7 Literature search results

**Figure 1:**
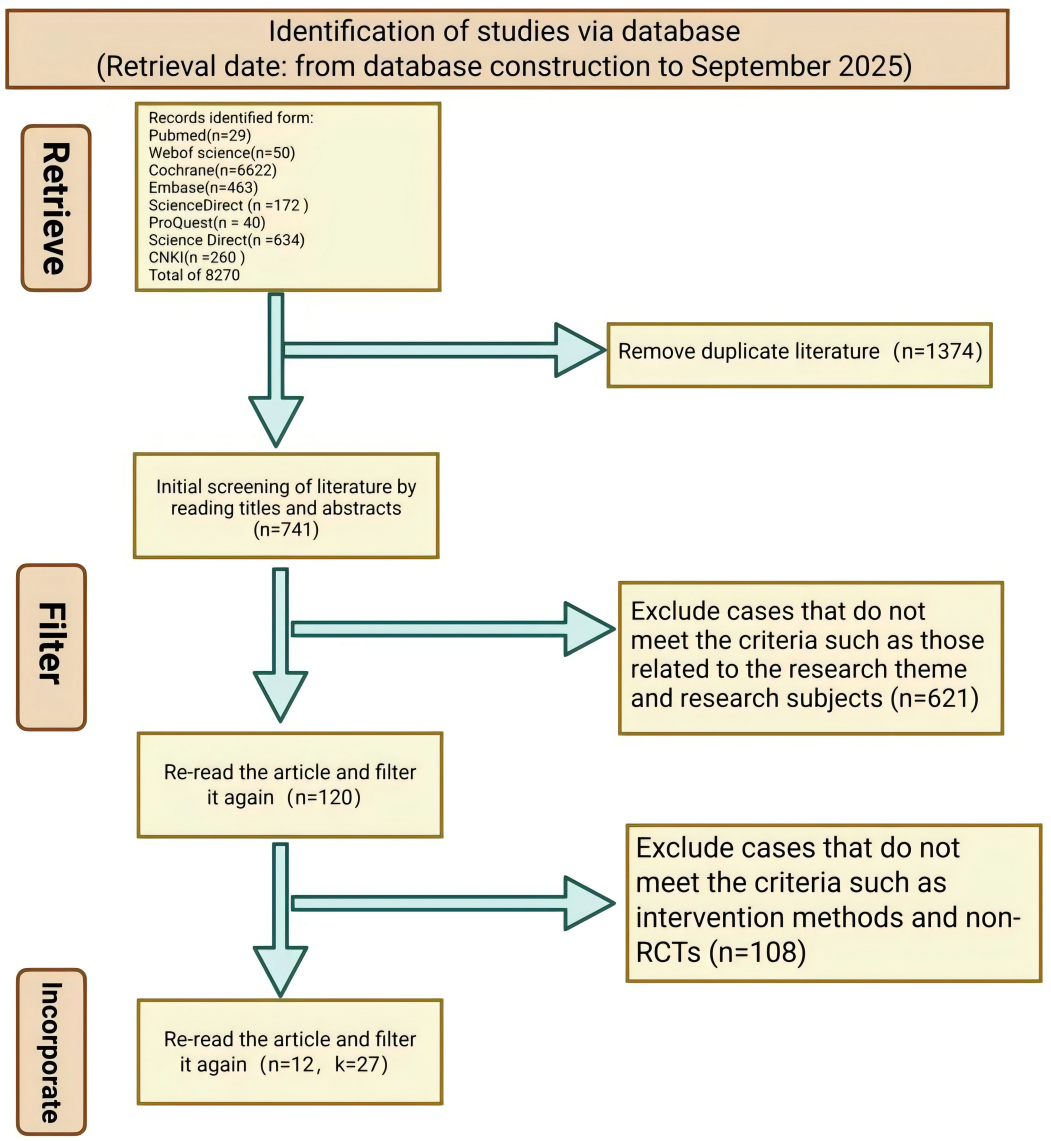
Literature search results.

The initial literature search identified 8,270 records. After removing 1,374 duplicates using EndNote X21, 6,896 articles were retained for title and abstract screening. This screening process led to the exclusion of 6,776 articles, resulting in 120 articles that underwent full-text review for eligibility. Following the full-text assessment, 108 articles were excluded, leaving 12 studies that met the inclusion criteria for qualitative synthesis and meta-analysis (**Figure 1**) (34–45).

### 2.8 Basic characteristics and quality assessment of the literature

This meta-analysis synthesized data from 12 studies involving 482 participants, which collectively yielded 27 effect sizes. The interventions were highly heterogeneous, including aerobic exercises, mind–body therapies, team sports, and skill-based physical activities. The risk of bias for each study was assessed using the Cochrane RoB tool, with three studies rated as low risk, seven as moderate risk, and two as high risk (see **Supplementary Material 3.2** for a detailed assessment).

### 2.9 Meta-analysis of the exercise interventions for children with ASD and comorbid conditions

#### 2.9.1 Overall effect analysis

A three-level random effects model was used to synthesize the 27 effect sizes from 12 studies, which evaluated the overall effect of exercise interventions on comorbid conditions, namely, anxiety, sleep disorders, and ADHD in children with ASD. The analysis revealed a large and statistically significant overall effect size (*g* = 0.879, 95%CI = 0.449–1.310, *p* < 0.001), indicating a strong positive effect of exercise interventions in this population (**Supplementary Material 3.3.1**). Substantial heterogeneity was observed among the included studies (*Q* = 146.65, *p* < 0.001), supporting the use of a random effects model.

#### 2.9.2 Sensitivity analysis

A leave-one-out sensitivity analysis was performed to assess the robustness of the pooled results. The overall effect size remained statistically significant (*p* < 0.01) after sequentially excluding each individual study, with point estimates ranging from *g* = 0.695 to *g* = 0. 914. Although the removal of one particular study [(44) (1)-2] resulted in the lowest effect size (*g* = 0.695, 95%CI = 0.438–0.951), the statistical significance and the direction of the overall effect were unchanged. These findings confirm the stability of the meta-analysis results.

#### 2.9.3 Heterogeneity analysis

A three-level variance decomposition model allocated 11.93% of the total variance to sampling error (level 1), 44.04% to within-study variance (level 2), and 44.04% to between-study variance (level 3). This distribution reveals substantial heterogeneity at both levels 2 and 3, indicating that the effect sizes varied not only across studies but also across different measurements within the same studies. These results support the use of a multilevel modeling framework and justify further investigation of the sources of heterogeneity through subgroup analysis or meta-regression.

#### 2.9.4 Publication bias assessment

This study employed systematic and comprehensive statistical analyses throughout the meta-analysis, underscoring its methodological rigor. Significant heterogeneity was observed among the included studies (*I*^2^ = 88.5%). We interpret this heterogeneity not as statistical noise but as a meaningful reflection of the varying effects of interventions across diverse real-world settings.

Examination of the funnel plot revealed asymmetry, while the Egger’s regression test indicated a significant risk of publication bias (*p* < 0.001). To evaluate the potential impact of this bias, a trim-and-fill analysis was performed. After imputing the potentially missing studies, the overall effect size remained statistically significant, suggesting that the primary conclusion is robust to potential publication bias (**Supplementary Material 3.3.4**).

### 2.10 Effects of exercise interventions on children with ASD

**Figure 2:**
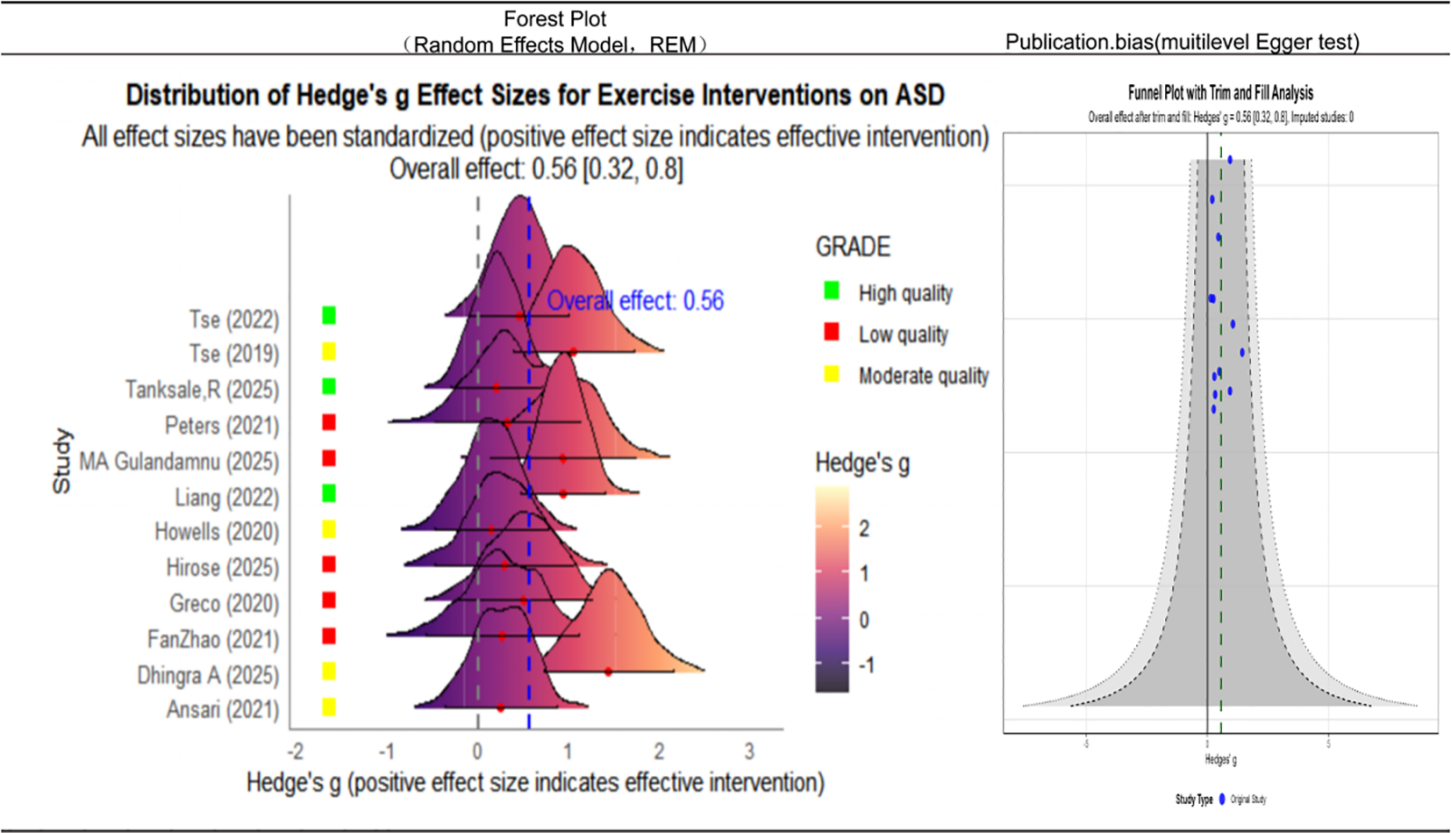
Forest plot and truncated funnel plot of the effects of exercise interventions on children with autism spectrum disorder (ASD). *Left*: Forest plot of the individual and pooled effect sizes (Hedges’ *g* and 95%CI). *Right*: Funnel plot for publication bias assessment (SE, standard error).

This study conducted a comprehensive evaluation of the effects of exercise interventions in children with ASD by incorporating data from 12 research studies (**Figure 2**). Using a random effects model to pool the effect sizes, the meta-analysis yielded an overall effect size of Hedge’s *g* = −0.56 (95%CI = 0.32–0.80, *p* < 0.001), including a moderate and statistically significant positive effect of physical exercise interventions in this population.

Heterogeneity testing revealed moderate heterogeneity across the studies (*I*^2^ = 37.5%, *Q* = 17.39), indicating that a portion of the variability in the effect sizes may be attributable to between-study differences. Nevertheless, the degree of heterogeneity was not high, supporting a reasonable level of consistency in the findings. To assess potential publication bias, the trim-and-fill method was applied. The results showed that no missing studies needed imputation, and both the effect size and the confidence intervals remained unchanged after adjustment (adjusted *g* = −0.56, 95%CI = 0.32–0.80). This indicates that publication bias had minimal influence and underscores the robustness of the findings.

Standardization of the effect directions addressed inconsistencies in reporting across some original studies (e.g., “lower scores” indicating improvement).

### 2.11 Effects of exercise interventions on comorbid anxiety in children with ASD

**Figure 3:**
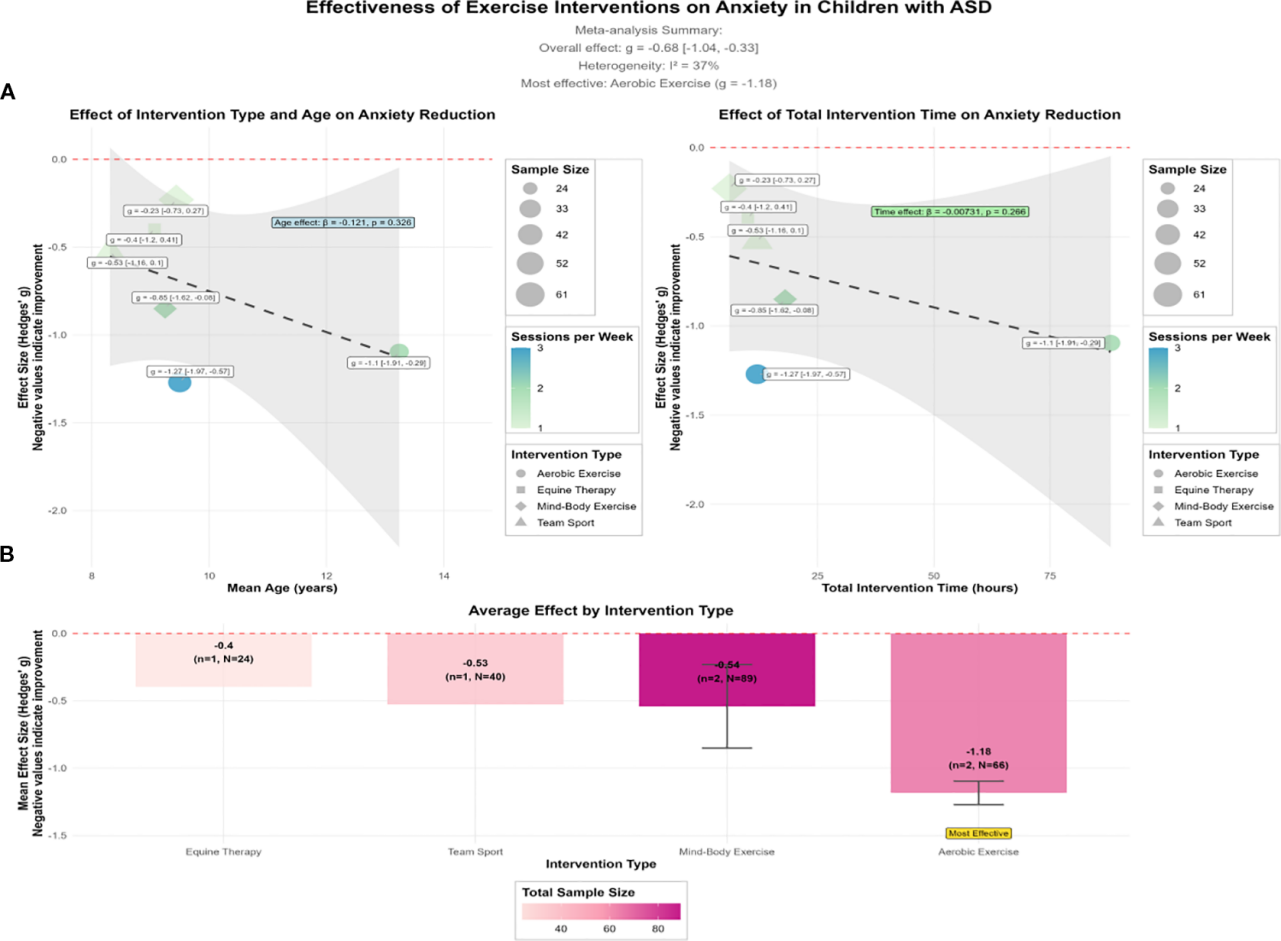
Analysis of the effect of exercise interventions on comorbid anxiety in children with autism spectrum disorder (ASD). Meta-regression plots depicting the associations of the effect size (Hedges’ *g*) with the intervention type and age **(A)** and the total intervention time **(B)**. The overall effect size, *I*^2^, *k* (number of studies), and *n* (sample size) are indicated.

This study comprehensively evaluated the efficacy of exercise interventions in comorbid anxiety in children with ASD by synthesizing data from six studies involving 219 participants (**Figure 3**). A random effects meta-analysis revealed a moderately large and statistically significant overall effect (*g* = −0.68, 95%CI = −1.04 to −0.33), indicating that exercise interventions are effective in reducing anxiety symptoms in this population. Heterogeneity among the included studies was low (*I*^2^ = 37%), and the findings remained relatively robust across sensitivity analyses.

Further analysis revealed that the effectiveness of the interventions was moderated by both the type of intervention and its dosage. Regarding the intervention type, aerobic exercise (e.g., dance therapy and aerobic training) demonstrated the largest anxiety-reducing effect (mean *g* = −1.18), followed by mind–body exercises (e.g., yoga and karate, *g* = −0.54) and team sports (*g* = −0.53). In contrast, animal-assisted therapy (e.g., equine therapy) was associated with a relatively smaller effect size (*g* = −0.40). These findings suggest that high-intensity aerobic exercise may offer greater benefits in emotion regulation and dissipation of anxious arousal.

In a meta-regression analysis examining the intervention dosage, a weekly frequency emerged as a significant moderator of effect size (*β* = −0.49, *p* = 0.009), suggesting that greater weekly exercise frequency is associated with a larger reduction in anxiety. In contrast, neither the total intervention duration (*p* = 0.266) nor the mean age of participants (*p* = 0.326) reached statistical significance, indicating that frequency may be a more influential moderator than cumulative intervention time. These findings lend support to a “short duration, high frequency” model for exercise-based interventions to alleviate anxiety.

### 2.12 Effects of exercise interventions on comorbid sleep disorders in children with ASD

**Figure 4:**
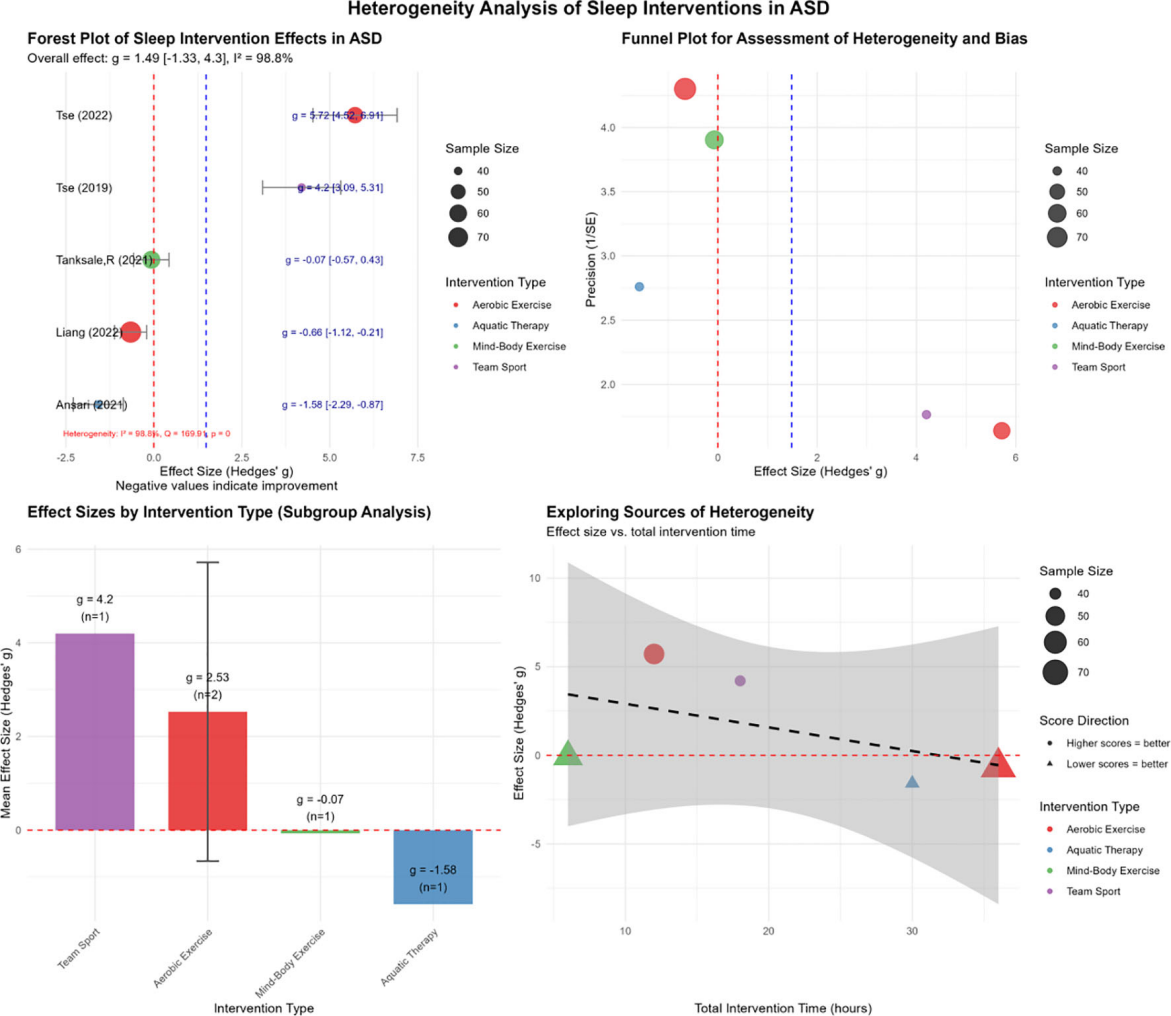
Analysis of the effects of exercise interventions on comorbid sleep disorders in children with autism spectrum disorder (ASD). Heterogeneity and meta-regression analysis for sleep outcomes displaying the overall effect size (Hedges’ *g* and 95% CI), *I*^2^, and *Q* statistic. The meta-regression results for total intervention time, age, and weekly frequency [moderators (QM) and *p*-value] are shown.

This study systematically evaluated the effects of exercise interventions on the sleep outcomes in individuals with ASD based on five studies involving a total of 274 participants (**Figure 4**). We further investigated the heterogeneity of the effect sizes and the potential moderating factors. Overall heterogeneity was assessed using Cochran’s *Q* test and the *I*^2^ statistic, which indicated substantial heterogeneity (*Q* = 169.91, *p* < 0.001; *I*^2^ = 98.8%). This suggests that the observed variation across studies was significantly greater than would be expected by chance alone, warranting further investigation into the potential sources of heterogeneity (**Figure 4**).

To investigate potential sources of heterogeneity, subgroup and meta-regression analyses were conducted. Subgroup analyses categorized the interventions by type (i.e., aerobic exercise, aquatic exercise, mind–body exercise, and team sports), which revealed variations in the effect sizes across intervention categories; however, the moderating effect of intervention type was not statistically significant (QM = 1.047, *p* = 0.79). Further meta-regression analyses were performed to assess the influence of continuous moderators—including mean age, total intervention duration, and weekly frequency—on the effect sizes. The results demonstrated that the total intervention duration had a significant moderating effect (QM = 16.265, *p* < 0.001) and accounted for a substantial proportion of the observed heterogeneity (*I*^2^ = 90%). In contrast, neither age (QM = 0.274, *p* = 0.601) nor weekly frequency (QM = 0.013, *p* = 0.91) exhibited a significant moderating effect. Outlier analysis based on Baujat plots indicated that individual studies substantially influenced both the overall heterogeneity and the model fit, suggesting the presence of potential outliers in the effect sizes or methodological variations (**Supplementary Material 3.6**).

### 2.13 Effects of exercise interventions on comorbid ADHD in children with ASD

**Figure 5:**
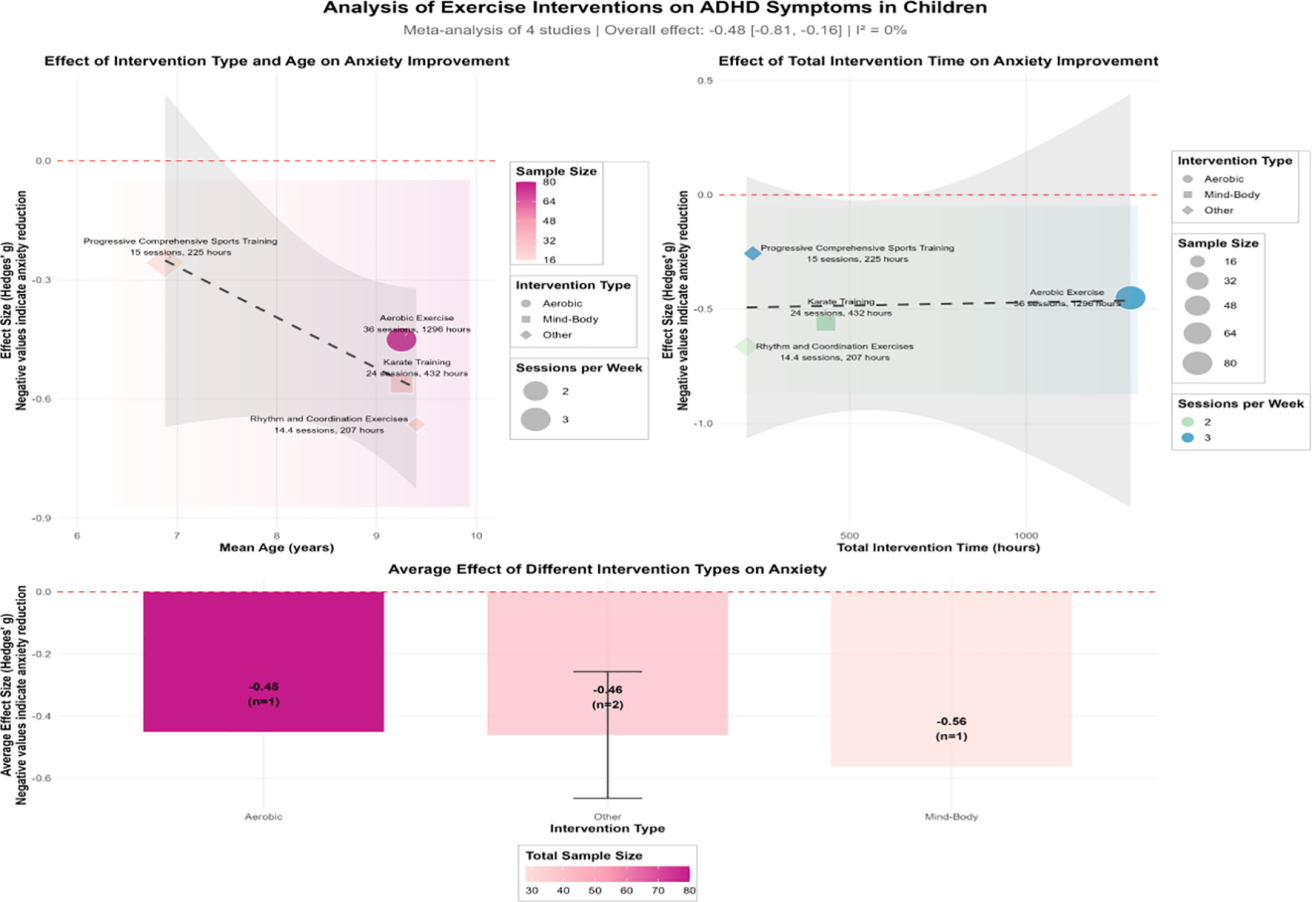
Analysis of the effect of exercise interventions on comorbid attention-deficit/hyperactivity disorder (ADHD) in children with autism spectrum disorder (ASD). Left: Forest plot of the individual and pooled effect sizes (Hedges’ *g* and 95% CI). Right: Relationship between effect size, intervention type, and age. The overall effect size, *I*^2^, *k*, and *n* are provided.

This study systematically evaluated the efficacy of exercise interventions in improving the core symptoms of ADHD in children. Four studies involving a total of 148 participants were included in the analysis (**Figure 5**).

The analysis revealed an overall effect size of *g* = −0.48 (95%CI = −0.81 to −0.16) for exercise interventions on ADHD symptoms, indicating a statistically significant, moderate beneficial effect. The tests of heterogeneity showed a non-significant Cochran’s *Q* statistic (*p > 0.05) and an *I*^2^ of 0%, suggesting minimal heterogeneity and consistent findings across the included studies. Descriptive analyses categorized by intervention type revealed some variation in the effect sizes. Relatively larger effects were observed for rhythm and coordination exercises (*g* = −0.66) and karate training (*g* = −0.56), whereas aerobic exercise (*g* = −0.45) and progressive combined training (*g* = −0.26) yielded more modest improvements. However, due to the limited number of available studies, these findings should be interpreted with caution. Based on the current evidence, exercise interventions appear to have a positive effect on the improvement of ADHD symptoms in children, with low heterogeneity across studies (*I*^2^ = 0%).

### 2.14 Multivariate meta-regression analysis of the training characteristics

**Figure 6:**
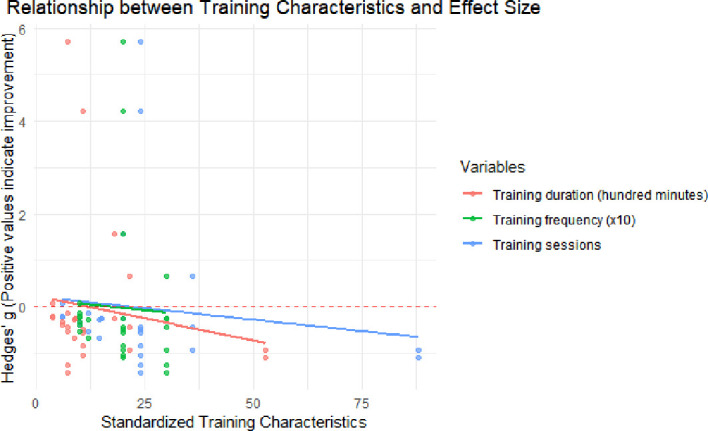
Multivariate meta-regression analysis of the training features. Multivariate meta-regression analyses of the associations between the number of sessions, session duration, weekly frequency, and the pooled effect size (Hedges’ *g*) displaying the regression coefficients with the QM (moderator) statistics and *p*-values.

This study utilized a mixed effects meta-regression model with restricted maximum likelihood estimation (REML) to assess the moderating effects of training frequency, single-session training duration, and weekly training frequency on the intervention effect size (Hedges’ *g*) (**Figure 6** and **Table 2**). The results indicated that the QM tests for all three moderators were non-significant: training frequency: QM (1) = 0.47, *p* = 0.495; training duration: QM (1) = 0.62, *p* = 0.432; and weekly training frequency: QM (1) = 0.07, *p* = 0.788. None of the regression coefficients reached statistical significance. The *R*^2^ value for each model was 0%, indicating that none of the training parameters accounted for the heterogeneity across studies. At the same time, the QE test revealed substantial residual heterogeneity (*I*^2^ > 94%, *p* < 0.0001). These results suggest that the training frequency, duration, and intensity are not the primary factors explaining the variation in the outcomes. Future research should explore other potential moderators, such as the intervention content quality, individual differences, and methodological features.

**Table 2:** Summary of the meta-analysis findings on the effects of exercise on the core autism spectrum disorder (ASD) symptoms and psychiatric comorbidities.

Outcome measure	No. of studies (*k*)	Sample size (*n*)	Effect size (Hedges’ *g*)	95%CI	Heterogeneity (*I*^2^)	Key moderator/finding
Overall core ASD symptoms	12	482	−0.56*	0.32–0.80	37.5% (moderate)	Significant overall improvement in the core symptoms
Comorbid anxiety	6	219	−0.68*	−1.04 to −0.33	37% (low)	Aerobic exercise showed the largest effect (*g* = −1.18). Frequency was a significant moderator (*p* = 0.009).
Comorbid sleep disorders	5	274	1.49	−1.33 to 4.30	98.8% (high)	Results inconclusive due to high heterogeneity. Intervention duration was a significant moderator (*p* < 0.001).
Comorbid ADHD	4	148	−0.48*	−0.81 to −0.16	0% (low)	Consistent beneficial effects. Rhythmic coordination exercises showed the largest effect (*g* = −0.66).

*k* denotes the number of studies and *n* the total no. of participants. For anxiety and ADHD, negative values indicate a reduction in symptom severity (improvement). For core ASD, positive values indicate an improvement (based on score standardization).

*CI*, confidence interval; *ADHD*, attention-deficit/hyperactivity disorder.

*p < 0.05 (statistically significant effect).

## 3 Discussion

This study employed a systematic search strategy across eight databases, initially identifying 8,270 relevant publications. After removing 1,374 duplicates, 6,896 articles were retained for further evaluation. Following a two-stage screening process based on the titles and abstracts and a subsequent full-text assessment, 12 eligible RCTs, encompassing a total of 482 participants, were ultimately included. This rigorous retrieval and selection process ensured comprehensive and systematic literature coverage, thereby establishing a robust foundation for subsequent analysis (46). Compared with existing reviews, this study addresses the broader clinical context in which high-functioning ASD (ASD-H) co-occur with anxiety, sleep disorders, and ADHD. For example, while Riis et al. (47) included eight studies focusing on anxiety interventions, the present review incorporates 12 studies on anxiety-related outcomes, reflecting broader inclusion criteria. The value of this comprehensive approach lies in its ability to capture a more representative picture of the comorbidity patterns of ASD; however, these insights remain constrained by the limited number of available studies, as also noted by Hossain et al. (48). The limited number of studies included in this review may be attributed to several factors, including the relatively recent emergence of research in this interdisciplinary area, the stringent inclusion criteria applied, and potential biases such as publication bias and language restrictions. The literature search results revealed a growing trend in the existing exercise interventions targeting comorbidities of ASD, such as anxiety, sleep disorders, and ADHD. Nevertheless, there remains a need for more high-quality RCTs to strengthen the evidence base. Consequently, the findings of this study can only be interpreted within the context of the available literature.

The 12 included studies involved a total of 482 children with ASD and employed a variety of intervention approaches, such as aerobic exercise, yoga combined with cognitive therapy, dance, martial arts, soccer, rhythmic balance training, aquatic activities, basketball, and equestrian therapy. Quality assessments rated three studies as high quality, seven studies as moderate quality, and two studies as low quality, suggesting that the overall evidence is generally acceptable despite some limitations. Although the diversity of interventions enriched insights into the efficacy of different exercise modalities, it also contributed to the increased heterogeneity in the results (49). Similar to the synthesis by Healy et al. (50) of 29 studies, the exercise interventions examined in this analysis showed moderate effects on symptom presentation in children with ASD. However, notable methodological variations were observed across the included studies, such as insufficient blinding procedures and heterogeneity in the control conditions (lack of blinding and inconsistent controls). The overall quality of the evidence may have been compromised by several sources of bias, including the small sample sizes, the challenges associated with the implementation of effective blinding, and the inconsistencies in the intervention duration and frequency (51). This suggests that the conclusions may be susceptible to certain biases related to the experimental design and reporting. For instance, the predominant reliance on subjective assessments by parents or teachers introduces the possibility of evaluator-dependent bias, which could affect the reliability of the outcomes. Consistent with this concern, individual studies have reported discrepancies among different observers, potentially undermining the consistency of the findings. While the overall evidence points to potential benefits of exercise interventions, methodological and implementation limitations indicate that the results should be interpreted with caution. Future research should focus on improving the study design to strengthen the validity and generalizability of the evidence.

A three-level random effects meta-analysis incorporating 12 studies (27 effect sizes) revealed that exercise interventions produced a moderate-to-large, statistically significant improvements in the core symptoms among children with ASD and comorbid anxiety, sleep disorders, or ADHD: Hedges’ *g* = 0.879 (95%CI = 0.449–1.310, *p* < 0.001). The sensitivity analysis further confirmed the robustness of the findings: sequentially removing each individual study yielded an effect size ranging from 0.695 to 0.914, with all estimates remaining statistically significant (*p* < 0.01) and without any change in the direction of the effects. These results are consistent with prior evidence, including a reported moderate overall effect size (*g* = 0.62) of exercise interventions in adolescents with ASD. Furthermore, other network meta-analyses have similarly demonstrated the beneficial effects of physical exercise on the social and behavioral outcomes in individuals with ASD. Statistically, the analysis revealed considerable heterogeneity (*I*^2^ = 88.5%), indicating substantial differences in the intervention protocols, measurement tools, and participant characteristics across the included studies. Therefore, a random effects model was adopted for the meta-analysis. Although Egger’s test suggested potential publication bias (*p* < 0.001), a trim-and-fill analysis demonstrated that the core findings remained consistent after imputing theoretically missing studies, thereby strengthening the reliability of the results (52). From a theoretical perspective, exercise may enhance neuroplasticity through the modulation of neurotransmitters—such as serotonin and norepinephrine—and the cortisol levels, which could in turn alleviate the core symptoms of ASD and related comorbidities (Liang et al., 2022). Nonetheless, the interpretation of these findings should be tempered by the presence of high heterogeneity and limited sample sizes. These limitations may introduce biases resulting from inconsistent intervention quality and non-uniform outcome measures. The overall findings should be interpreted in conjunction with the context of the individual studies included (53). This study provides robust evidence supporting the value of exercise interventions as an adjunct therapeutic approach, establishing a solid foundation for future detailed subgroup and dose–response analyses. In the sub-analyses focusing on the core symptoms of children with ASD, the exercise interventions demonstrated a moderate effect on ASD symptoms (Hedges’ *g* = −0.56, 95%CI = 0.32–0.80, *p* < 0.001). The heterogeneity analysis revealed a moderate level of variation across the included studies (*I*^2^ = 37.5%, *Q* = 17.39), with a high degree of statistical consistency. The trim-and-fill meta-analysis indicated no need for adjustment, suggesting limited publication bias. These results support the conclusion that exercise interventions can produce moderate improvements in the core symptoms of ASD in children, including social communication and repetitive behaviors. This finding is consistent with prior research, such as the study by Woodman et al. (54), which reported that high-intensity exercise significantly reduces repetitive behaviors in children with ASD. Yano et al. (55) further proposed that exercise interventions act as a key mechanism linking the immune, muscular, and brain systems, leading to improvements in social communication and a reduction in repetitive behaviors among children with ASD. From a mechanistic standpoint, exercise may enhance neural function by elevating the levels of brain-derived neurotrophic factor (BDNF) and modulating connectivity within neural networks. Concurrently, exercise interventions have been shown to promote beneficial changes in white matter connectivity and to enhance executive function networks in children with ASD (56, 57). Overall, this study supports the use of exercise as an adjunctive intervention for improving the core symptoms of ASD and provides preliminary evidence to further elucidate the mechanisms underlying different exercise modalities (58).

In the subgroup analysis of children with ASD with comorbid anxiety symptoms (six studies, 219 participants), exercise interventions were associated with a significant reduction in anxiety levels, demonstrating a moderately large effect size (Hedges’ *g* = −0.68, 95%CI = −1.04 to −0.33, *p* < 0.001). Similar to the analysis of the core ASD symptoms, the negative effect size indicates a significant reduction in anxiety symptoms. The heterogeneity among the studies was low (*I*^2^ = 37%), supporting the robustness of the findings. Subgroup analyses revealed variations in anxiety reduction according to exercise type: aerobic exercise (e.g., dance and aerobic training) demonstrated the largest effect size (mean effect size = 1.18). Our subgroup analysis specifically highlights that aerobic exercise is superior to other modalities for anxiety reduction. Clinically, this suggests that interventions aiming to reduce physiological arousal in anxious children with ASD should prioritize continuous, rhythmic aerobic activities over complex motor skills training, followed by mind–body exercises (e.g., yoga and karate, *g* ≈ −0.54) and team sports (*g* = 0.53), whereas animal-assisted therapy (e.g., equine therapy) was associated with a relatively smaller effect (*g* = 0.40). Meta-regression further indicated that a weekly intervention frequency was a significant moderator of anxiety improvement, with higher frequencies associated with greater reductions in anxiety (*β* = −0.49, *p* = 0.009). In contrast, neither the total intervention duration nor the participant age reached statistical significance. This finding indicates that exercise programs characterized by a short duration and high frequency may be particularly beneficial in reducing anxiety among children with ASD. The anxiolytic effects of exercise are likely mediated through multiple physiological and psychological mechanisms, including regulation of the hypothalamic–pituitary–adrenal (HPA) axis, the release of endorphins, and the overall mood enhancement. Additional contributing factors may involve the stabilization of daily routines and improvements in self-efficacy (59, 60). These results are consistent with the systematic review by Trzmiel et al. (61), which supports the efficacy of various forms of physical activity—such as yoga, soccer, group-based sports, and equestrian therapy—in alleviating anxiety symptoms in individuals with ASD (3, 40, 62).

This study, which included five randomized trials involving a total of 274 children with ASD, evaluated the effects of intervention on comorbid sleep disorders. The results revealed extreme heterogeneity across studies (Cochran’s *Q* = 169.91, *p* < 0.001, *I*^2^ = 98.8%), which compromised the reliability of the pooled effect estimate (*g* = 1.49, 95%CI = −1.33 to 4.30, including zero). Subgroup analysis based on exercise type revealed no statistically significant differences (QM = 1.047, *p* = 0.79), suggesting inconsistent—but not statistically significant—effects across intervention types. The meta-regression analysis revealed that the total intervention duration was the primary source of heterogeneity (QM = 16.265, *p* < 0.001, *I*^2^ = 90%), suggesting that longer exercise interventions are generally associated with greater sleep benefits. In contrast, neither the mean age nor the exercise frequency demonstrated a significant association with the outcomes. The observed heterogeneity may be attributed to the inconsistencies across studies regarding the assessment criteria for sleep disorders, the assessment methods (e.g., subjective sleep questionnaires *vs*. objective monitoring), and the variations in the intervention content (63–65). The limited sample sizes and the considerable heterogeneity in the data constrained the statistical power of the analysis; therefore, caution is advised against overinterpreting the subgroup findings (66). Given the extreme heterogeneity (*I*^2^ > 90%) and the wide confidence intervals, current evidence remains inconclusive with regard to the effect of physical exercise on sleep disorders in children with ASD (67). Consequently, future research should aim to enroll larger cohorts, employ standardized assessment protocols, and further investigate underlying mechanisms—such as the influence of exercise on the circadian rhythm—to establish more conclusive outcomes (68).

The subgroup analysis of children with ASD with comorbid ADHD symptoms (four studies, 148 participants) showed a pooled effect size of Hedges’ *g* = −0.48 (95%CI = −0.81 to −0.16, *p* < 0.01), indicating a moderate reduction in the core ADHD symptoms following exercise interventions. Heterogeneity was low (*I*^2^ = 0%), suggesting consistent results across the included studies without significant statistical heterogeneity. Among the included studies, the effect sizes varied according to the exercise modality: rhythmic coordination activities (e.g., soccer) yielded the greatest effect (*g* = −0.66). This finding has significant clinical relevance, implying that exercises requiring cognitive engagement and complex motor planning (e.g., team sports and martial arts) are more effective at addressing executive function deficits than simple repetitive movements, followed by karate training (*g* = −0.56). In contrast, aerobic exercise (*g* = −0.45) and progressive combined training (*g* = −0.26) were associated with relatively smaller effects. This pattern may be attributable to the cognitive demands inherent in rhythm-based and team sports, which are known to engage attention and executive function (69). These results are consistent with the findings of Seiffer et al. (70), who observed small to moderate effects of regular moderate-to-vigorous exercise on ADHD symptoms (*g* = −0.33). Together, these findings support the role of exercise as an adjunctive therapeutic intervention in ADHD.

A mixed effects meta-regression analysis was conducted to assess the moderating effects of training volume, single-session duration, and weekly frequency on the overall effect size. None of these moderators reached statistical significance: the training volume (QM = 0.47, *p* = 0.495), single-session duration (QM = 0.62, *p* = 0.432), and weekly frequency (QM = 0.07, *p* = 0.788) did not significantly influence treatment efficacy. Each regression coefficient was non-significant, with a model *R*^2^ = 0, indicating that these training dose metrics could not explain the heterogeneity across studies. Concurrently, the residual heterogeneity remained exceptionally high (*I*^2^ > 94%, *p* < 0.0001), suggesting that additional variables likely contributed to the observed differences in the effects. These findings imply that quantifying the efficacy of the exercise interventions based solely on the training frequency or the duration is challenging, possibly due to more complex factors such as exercise quality, type specificity, or individual participant characteristics (71). Previous studies have frequently highlighted the significance of exercise frequency and intensity—as reflected, for instance, in the anxiety group outcomes of this study, underscoring the role of frequency. However, the multivariate regression analysis conducted at the overall sample level did not reveal significant associations (72). The limited sample size (12 studies) may have been insufficient to support complex regression modeling. Furthermore, the observed relationships suggest that the effects of exercise prescriptions are not simply linearly incremental but may involve threshold effects or interaction mechanisms (73). Due to the limitations of the available data, this study cannot confirm that specific training scale parameters exert universal moderating effects. Future research should focus on more nuanced moderating variables, such as the quality of the intervention content, social factors, and individual baseline characteristics (74).

The positive effects of exercise on anxiety and psychiatric comorbidities in children with ASD may be mediated by several neurobiological mechanisms. Firstly, regarding anxiety reduction, physical activity acts as a physiological stressor that helps regulate the HPA axis, leading to reduced basal cortisol levels and improved stress resilience. Crucially, aerobic exercise has been shown to dampen the hyperactivity of the amygdala—the brain’s fear center often dysregulated in ASD—thereby modulating emotional reactivity (49). Secondly, for ADHD-related symptoms, exercise stimulates the secretion of monoamine neurotransmitters, such as dopamine and serotonin, which play pivotal roles in mood regulation and attentional control (69). Specifically, complex motor activities (e.g., rhythmic or team sports) may activate the prefrontal cortex–cerebellum loop, which underpins executive function and inhibitory control. Furthermore, regular physical activity has been shown to upregulate BDNF, facilitating neuroplasticity and optimizing brain network connectivity, which are often dysregulated in children with ASD (49, 75).

This study offers systematic evidence regarding the efficacy of exercise interventions for children with ASD and co-occurring psychiatric conditions. However, several limitations must be noted. Firstly, the majority of the included studies are single-center trials with small sample sizes. While small sample sizes in primary studies increase the risk of type II errors, this meta-analysis mitigates that limitation by synthesizing the data into a larger pooled sample (*N* = 482), thereby enhancing the statistical power to detect significant treatment effects. Nevertheless, the generalizability of the findings may still be constrained by the variability in the intervention protocols (76). Secondly, the reliance on subjective assessment tools may introduce detection bias due to inter-rater variability (77). In particular, findings related to sleep outcomes are less reliable owing to measurement heterogeneity (78). Thirdly, signs of publication bias were detected (Egger’s test, *p* < 0.001). Although we actively engaged with grey literature (e.g., ProQuest and CNKI) to evaluate potential biases—as recommended in recent guidelines (79, 80)—the risk of bias persists. However, the trim-and-fill analysis demonstrated that the core findings remained consistent after imputing theoretically missing studies, suggesting that our primary conclusions are robust despite this potential limitation (5, 29). To advance the field, subsequent research should prioritize large-scale, multicenter RCTs and combine subjective questionnaires with objective physiological measures—such as actigraphy or neuroimaging—to reduce assessment bias (81). Furthermore, future research should prioritize elucidating the neurobiological mechanisms through which exercise exerts its effects, such as the modulation of brain network dynamics (82).

In light of current evidence, specific clinical recommendations can be formulated. For individuals with ASD and co-occurring anxiety, prioritizing moderate-to-high intensity aerobic exercise is recommended (83). For those with attention deficits (ADHD symptoms), interventions emphasizing rhythmic coordination activities and structured team sports may be particularly beneficial (84). Comprehensive reporting of intervention parameters—including heart rate zones and social context—will further facilitate dose–response analysis in clinical practice (85).

## 4 Conclusions

This study systematically evaluated the evidence-based effects of exercise interventions on the core symptoms of children with ASD with comorbid anxiety and ADHD while comparing outcomes across different exercise modalities. The results partially supported our hypotheses: overall, exercise interventions produced beneficial therapeutic effects, with aerobic exercise yielding the greatest reduction in anxiety symptoms and rhythmic coordination exercises demonstrating stronger regulatory effects on attention control. The core contribution of this study lies in being the first to analyze children with comorbid ASD and anxiety alongside ADHD as a distinct clinical subgroup, elucidating the specific benefits of various forms of exercise across different diagnostic dimensions. Based on the accumulated evidence, we recommend implementing a combined intervention for this population. Specifically, a “FITT” (frequency, intensity, time, and type of exercise) framework is suggested for consideration based on current evidence: *frequency* of three to four sessions per week; *intensity* at moderate-to-vigorous levels; *time* ranging from 30 to 60 min per session; and *type* focusing on aerobic exercise (for anxiety reduction) combined with rhythm-based team sports (for attention regulation). The subgroup analyses further supported this model, indicating that high-frequency interventions yield superior outcomes compared with low-frequency regimens.

## Data Availability

The original contributions presented in the study are included in the article/**Supplementary Material**. Further inquiries can be directed to the corresponding author.
