# Supplementary material for: Effects of exercise on anxiety and psychiatric comorbidities in children with autism spectrum disorder: a systematic review and meta-analysis
Source: Front Psychiatry. 2026 Feb 20;17:1761833. doi: 10.3389/fpsyt.2026.1761833 (PMC12963353; doi:10.3389/fpsyt.2026.1761833)

**Supplementary Materials**

2.4 Basic Characteristics and Quality Assessment of Literature

| Author/Year | Country | Sample Size (Experimental/Control) | Mean Age ± SD | Gender Ratio (Male/Female) | Intervention Type | Intervention Frequency & Duration | Outcome Measurement Tool | Intervention Time Points | Outcome Indicators |
| --- | --- | --- | --- | --- | --- | --- | --- | --- | --- |
| Dhingra (2025) | India | 38 (19/19) | Group A: 11 (range 8-13); Group B: 8 (range 7-12) | Both groups: 18:1 | Aerobic Training | 30 minutes/session, 3 sessions/week, total 8 weeks | SCARED, FIM | Baseline, Week 8 | ①③ |
| Tanksale (2025) | Australia | 61 (31/30) | 9.42±1.34 / 9.46±1.38 | IE: 21:10; WLC: 18:12 | Yoga + Third-Wave CBT | 60 minutes/session, 1 session/week, total 6 weeks | BRIEF-2, CSHQ, ASC-ASD, EAQ-30, GAS | Baseline, Post-intervention, 6-week Follow-up | ①②③ |
| MA (2025) | China | 28 (17/11) | 13.29±1.80 / 13.18±1.60 | IE: 12:5; CG: 10:1 | Dance/Movement Therapy | 45–60 minutes/session, 2 sessions/week, total 11 months (88 sessions) | ABC, RBS-R, SRS, SCQ | Baseline, Post-intervention (Month 11) | ①③ |
| Tse (2021) | Hong Kong, China | 55 (23/32) | 10.86±1.99 / 11.17±1.93 | IE: 21:2; CG: 26:6 | Morning Jogging (Aerobic) | 30 minutes/session, 2 sessions/week, total 12 weeks (24 sessions) | Actigraphy, Sleep Diary, Urinary aMT6s, GARS-3 | Baseline, Post-intervention, 6-week Follow-up | ①② |
| Greco (2020) | Italy | 14 / 14 | 9.25 ± 1.00 | 24:4 (total) | Karate (Kata) | 2 sessions/week, 45 minutes/session, 12 weeks | SSIS-RS, BRIEF | Baseline, After Week 12 | ①③④ |
| Howell (2020) | Australia | 19 / 21 | 8.31 ± 2.01 | 37:3 (total) | Football (Auskick) | 1 session/week, 60–90 minutes/session, average 12 sessions | CBCL, VABS-3 | Baseline, After Weeks 6–8 | ①③ |
| Hirose (2025) | Japan | ASD:15, ADHD:12 | 9.4 ± 1.8 (total) | ASD:13:2, ADHD:8:4 | Rhythm/Balance/Coordination Training | Average 1.2 sessions/week, 12 weeks/60 minutes/session | SRS-2, Conners 3 | Baseline, Weeks 4, 8, 12 | ①④ |
| Ansari (2021) | Iran | 20 / 20 | 11.10 ± 2.01 | All male | Water Sports | 2 sessions/week, 60 minutes/session, 10 weeks | CSHQ | Baseline, After Week 10 | ② |
| Tse (2022) | Hong Kong, China | 40 (19/21) | 9.95 | Not specified | Basketball Skill Learning | 45 minutes/2 sessions/12 weeks | Actigraphy, Sleep Diary, Go/No-Go (GNG) Task, etc. | Baseline, After Week 12 | ①② |
| Liang (2022) | Hong Kong, China | 80 (EG:40, CG:40) | 8.46 ± 1.50 years | EG:30:10, CG:32:8 | Aerobic Exercise | 3 times/week, 60 minutes/session, total 12 weeks | Flanker Task, Tower of London, TMT, PSQI, PAQC | Pre-test, Post-test, 12-week Follow-up | ①②④ |
| Zhao Fan (2021) | China | 17 (Intervention) / 8 (Control) | 6.88 ± 3.05 years | 19:6 (total) | Comprehensive Sports Training | 3 times/week, 60 minutes/session, total 5 weeks (15 sessions) | ABC Scale, Hyperactivity Observation Form, Heart Rate Monitoring | Pre-test, Mid-test, Post-test, 1-month Follow-up | ①④ |
| Peters (2021) | United States | 11/9 | Experimental group: 8.68±2.09; Control group: 9.45±1.62 | Experimental group:10/2; Control group:6/3 | Equestrian Therapy | 60 minutes/1 session/week, total 10 weeks | GAS, ABC-C, SRS-2, PEDI-CAT ASD, Hair Cortisol Concentration (HCC) | Baseline, 10 weeks post-intervention | ①③ |

Note: ①: ASD (Autism Spectrum Disorder); ②: Sleep Disorders; ③: Anxiety; ④: ADHD (Attention Deficit Hyperactivity Disorder) Intervention Measures

3.2 Basic Characteristics and Quality Assessment of Literature

| study | year | intervention | n_E | n_C | mean_post_C | sd_post_C | mean_post_E | sd_post_E | outcome | direction | sessions | duration_min | freq_week |
| --- | --- | --- | --- | --- | --- | --- | --- | --- | --- | --- | --- | --- | --- |
| Ansari-1 | 2021 | Movement in water | 20 | 20 | 60.8 | 6.32 | 51.85 | 4.65 | Sleep | reduce | 20 | 1800 | 2 |
| Ansari-2 | 2021 | Movement in water | 20 | 20 | 47.75 | 9.21 | 45.35 | 9.87 | ASD | reduce | 20 | 1800 | 2 |
| Dhingra A-1 | 2025 | Aerobic Training | 19 | 19 | 92 | 16.5 | 111 | 8 | ASD | enhance | 24 | 720 | 3 |
| Dhingra A-2 | 2025 | Aerobic Training | 19 | 19 | 49.75 | 6.67 | 42.75 | 3.7 | Anxiety | reduce | 24 | 720 | 3 |
| Greco-1 | 2020 | Karate Training | 14 | 14 | 75.9 | 7.4 | 71.9 | 8.3 | ASD | reduce | 24 | 1080 | 2 |
| Greco-2 | 2020 | Karate Training | 14 | 14 | 76.4 | 7.2 | 71.9 | 8.3 | ADHD | reduce | 24 | 1080 | 2 |
| Greco-3 | 2020 | Karate Training | 14 | 14 | 120.3 | 10.7 | 111.6 | 9.1 | Anxiety | reduce | 24 | 1080 | 2 |
| Hirose-1 | 2025 | Rhythm and Coordination Exercises | 15 | 12 | 77.3 | 22 | 71.3 | 18.7 | ASD | reduce | 14.4 | 864 | 1.2 |
| Hirose-2 | 2025 | Rhythm and Coordination Exercises | 12 | 12 | 14.6 | 4.9 | 10.6 | 6.6 | ADHD | reduce | 14.4 | 864 | 1.2 |
| Howells-1 | 2020 | Community Football Program | 21 | 19 | 67.38 | 7.43 | 66.26 | 8.17 | ASD | reduce | 12 | 720 | 1 |
| Howells-2 | 2020 | Community Football Program | 21 | 19 | 66.67 | 11.76 | 60.95 | 9.54 | Anxiety | reduce | 12 | 720 | 1 |
| Liang-1 | 2022 | Aerobic Exercise | 39 | 39 | 26.51 | 3.4 | 29.54 | 3.01 | ASD | enhance | 36 | 2160 | 3 |
| Liang-2 | 2022 | Aerobic Exercise | 39 | 39 | 6.92 | 3.29 | 5.03 | 2.28 | Sleep | reduce | 36 | 2160 | 3 |
| Liang-3 | 2022 | Aerobic Exercise | 40 | 40 | 927.23 | 397.18 | 771.59 | 275.8 | ADHD | reduce | 36 | 2160 | 3 |
| MA Gulandamnu-1 | 2025 | Dance Therapy | 17 | 11 | 4.47 | 1.59 | 3.06 | 1.39 | ASD | reduce | 88 | 5280 | 2 |
| MA Gulandamnu-2 | 2025 | Dance Therapy | 17 | 11 | 2.55 | 1.21 | 1.59 | 0.51 | Anxiety | reduce | 88 | 5280 | 2 |
| Peters-1 | 2021 | Equine Therapy | 12 | 12 | 14.65 | 6.99 | 12 | 5.89 | Anxiety | reduce | 10 | 600 | 1 |
| Peters-2 | 2021 | Equine Therapy | 12 | 12 | 73.7 | 8.51 | 71.1 | 7.15 | ASD | reduce | 10 | 600 | 1 |
| Tanksale,R-1 | 2025 | Yoga | 31 | 30 | 74.7 | 7.29 | 73.19 | 7.75 | ASD | reduce | 6 | 360 | 1 |
| Tanksale,R-2 | 2021 | Yoga | 31 | 30 | 46.97 | 7.06 | 46.42 | 7.96 | Sleep | reduce | 6 | 360 | 1 |
| Tanksale,R-3 | 2022 | Yoga | 31 | 30 | 25.03 | 13.51 | 21.97 | 12.74 | Anxiety | reduce | 6 | 360 | 1 |
| Tse（1）-1 | 2022 | Morning Jogging | 23 | 32 | 18.8 | 6.6 | 15.33 | 8.73 | ASD | reduce | 24 | 720 | 2 |
| Tse（1）-2 | 2022 | Morning Jogging | 23 | 32 | 89.6 | 1.13 | 94.8 | 0.37 | Sleep | enhance | 24 | 720 | 2 |
| Tse（2）-1 | 2019 | Basketball Skill Training | 19 | 21 | 18.05 | 7.8 | 10.89 | 5.17 | ASD | reduce | 24 | 1080 | 2 |
| Tse（2）-2 | 2019 | Basketball Skill Training | 19 | 21 | 90.73 | 1.67 | 96.33 | 0.71 | Sleep | enhance | 24 | 1080 | 2 |
| FanZhao-1 | 2021 | Progressive Comprehensive Sports Training | 17 | 8 | 49.5 | 17.53 | 43.53 | 23.81 | ASD | reduce | 15 | 900 | 3 |
| FanZhao-2 | 2021 | Progressive Comprehensive Sports Training | 8 | 8 | 3.14 | 1.46 | 2.83 | 0.69 | ADHD | reduce | 15 | 900 | 3 |

Note C: Control group E: Experimental group


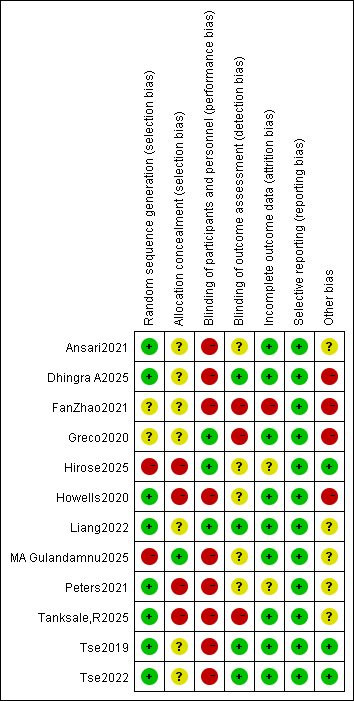


3.3.1 Meta-Analysis of Exercise Interventions for Children with Autism Spectrum Disorder (ASD) Comorbid with Anxiety, Sleep Disorders, or Attention-Deficit/Hyperactivity Disorder (ADHD)

Sensitivity Analysis

| study_removed | estimate | ci_lb | ci_ub | p_value |
| --- | --- | --- | --- | --- |
| Ansari-1 | 0.853564502 | 0.40825135 | 1.298877653 | 0.000566647 |
| Ansari-2 | 0.90624883 | 0.45855789 | 1.353939771 | 0.000320602 |
| Dhingra A-1 | 0.859652154 | 0.41234236 | 1.306961948 | 0.000551724 |
| Dhingra A-2 | 0.866257888 | 0.417163473 | 1.315352302 | 0.000531489 |
| Greco-1 | 0.896253818 | 0.446765522 | 1.345742114 | 0.000376623 |
| Greco-2 | 0.893711012 | 0.443828136 | 1.343593888 | 0.000391702 |
| Greco-3 | 0.882858694 | 0.432351955 | 1.333365433 | 0.000451574 |
| Hirose-1 | 0.903870417 | 0.456287976 | 1.351452858 | 0.000328923 |
| Hirose-2 | 0.889680391 | 0.439759897 | 1.339600884 | 0.000411119 |
| Howells-1 | 0.910155556 | 0.463914863 | 1.356396248 | 0.000295508 |
| Howells-2 | 0.895842842 | 0.445549982 | 1.346135702 | 0.000385671 |
| Liang-1 | 0.879671822 | 0.427585637 | 1.331758006 | 0.000486039 |
| Liang-2 | 0.890906753 | 0.439044525 | 1.342768981 | 0.000423915 |
| Liang-3 | 0.899385065 | 0.448848805 | 1.349921326 | 0.000372031 |
| MA Gulandamnu-1 | 0.880855263 | 0.430519481 | 1.331191045 | 0.000460519 |
| MA Gulandamnu-2 | 0.879767362 | 0.429489107 | 1.330045617 | 0.00046583 |
| Peters-1 | 0.899474546 | 0.4510169 | 1.347932191 | 0.000353827 |
| Peters-2 | 0.902217833 | 0.454438468 | 1.349997198 | 0.000337021 |
| Tanksale,R-1 | 0.908899522 | 0.461518969 | 1.356280075 | 0.000308354 |
| Tanksale,R-2 | 0.913674162 | 0.468317572 | 1.359030752 | 0.000277312 |
| Tanksale,R-3 | 0.907685955 | 0.459856452 | 1.355515458 | 0.000316232 |
| Tse（1）-1 | 0.899680088 | 0.449680763 | 1.349679414 | 0.000366082 |
| Tse（1）-2 | 0.694618066 | 0.438052207 | 0.951183925 | 8.47E-06 |
| Tse（2）-1 | 0.874193518 | 0.423571981 | 1.324815056 | 0.000501268 |
| Tse（2）-2 | 0.754045876 | 0.402260804 | 1.105830948 | 0.000169926 |
| FanZhao-1 | 0.902815116 | 0.455467217 | 1.350163015 | 0.000331203 |
| FanZhao-2 | 0.90287872 | 0.456534066 | 1.349223375 | 0.000323072 |

Note: study_removed: Excluded studies estimate: Pooled effect size estimate ci_lb: Lower confidence interval bound p_value: P-value

Heterogeneity Analysis

| Level | Variance | Percentage |
| --- | --- | --- |
| Level 1 (Sampling Error) | 0.141605212 | 11.92923693 |
| Level 2 (Within Research) | 0.522719061 | 44.03538154 |
| Level 3 (Research Room) | 0.522719061 | 44.03538154 |

Overall Effect Size

| Model Type | Effect size | Standard deviation | CI lower limit | CI upper limit | p-value | Q-value | Q_p value |
| --- | --- | --- | --- | --- | --- | --- | --- |
| Three-Level Model | 0.879445288 | 0.20928349 | 0.449256913 | 1.309633664 | 0.00027547 | 146.6516579 | 8.59E-19 |
| Two-level model | 0.879445288 | 0.209283489 | 0.449256916 | 1.309633659 | 0.00027547 | 146.6516579 | 8.59E-19 |

3.3.4 Publication Bias Assessment
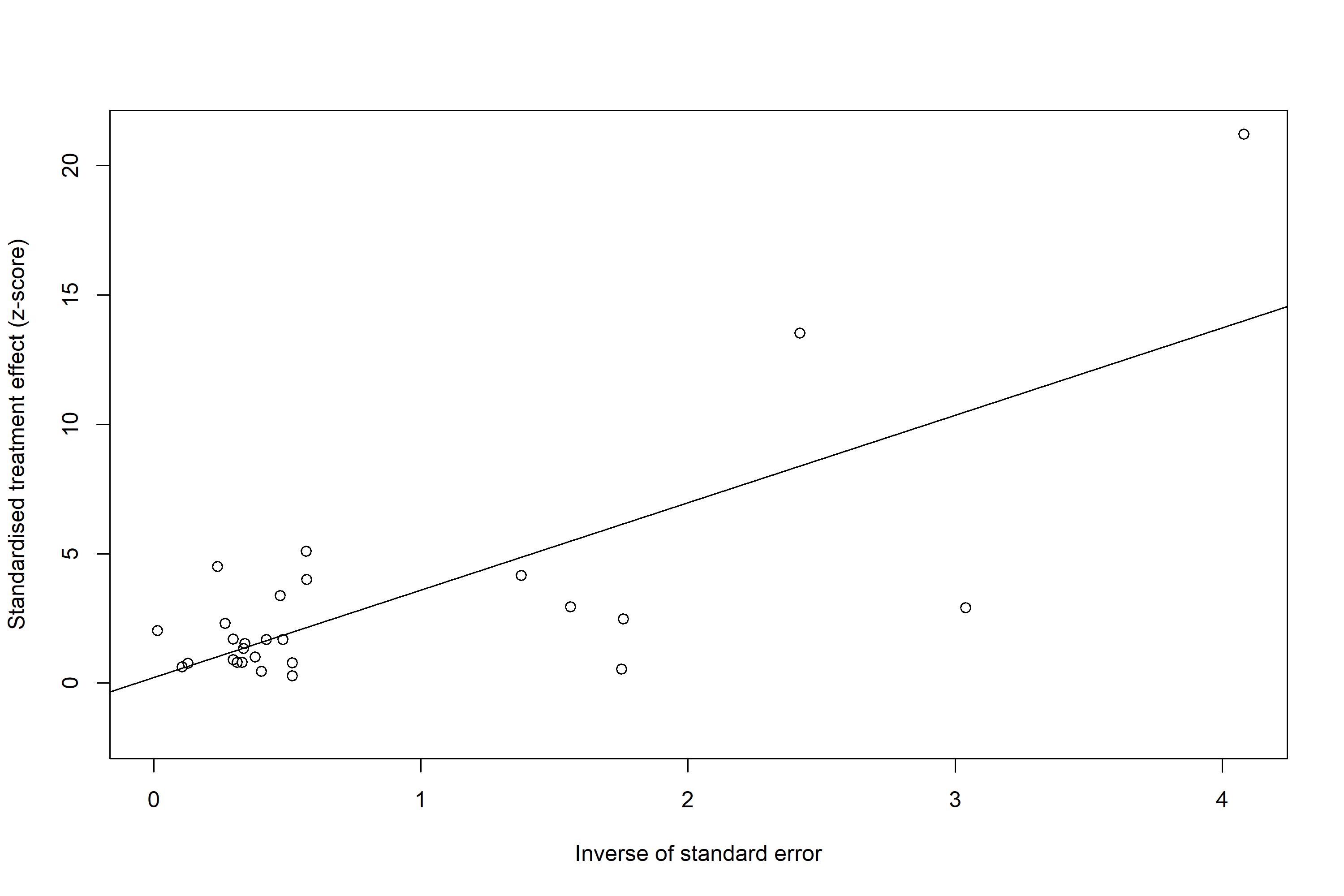

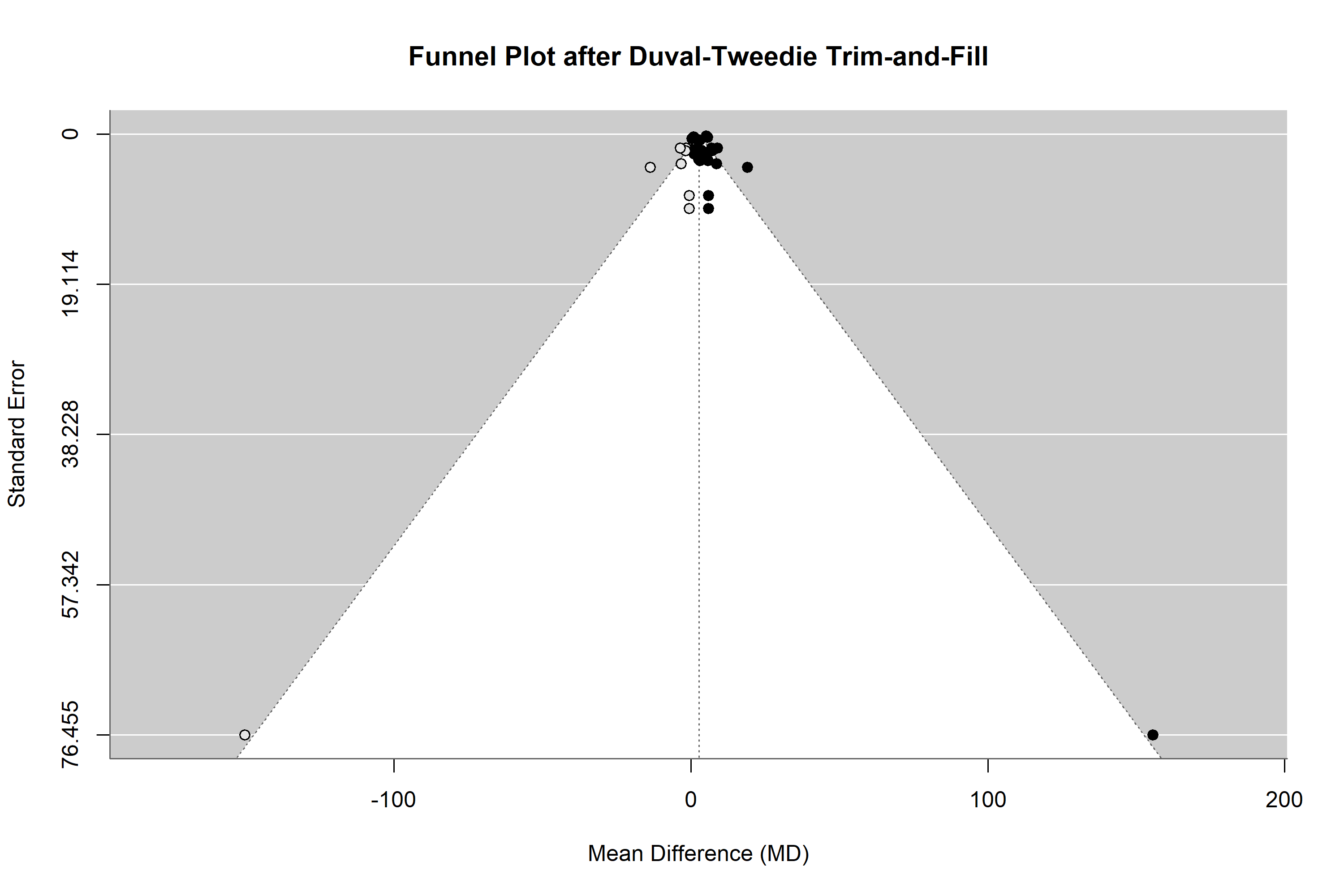

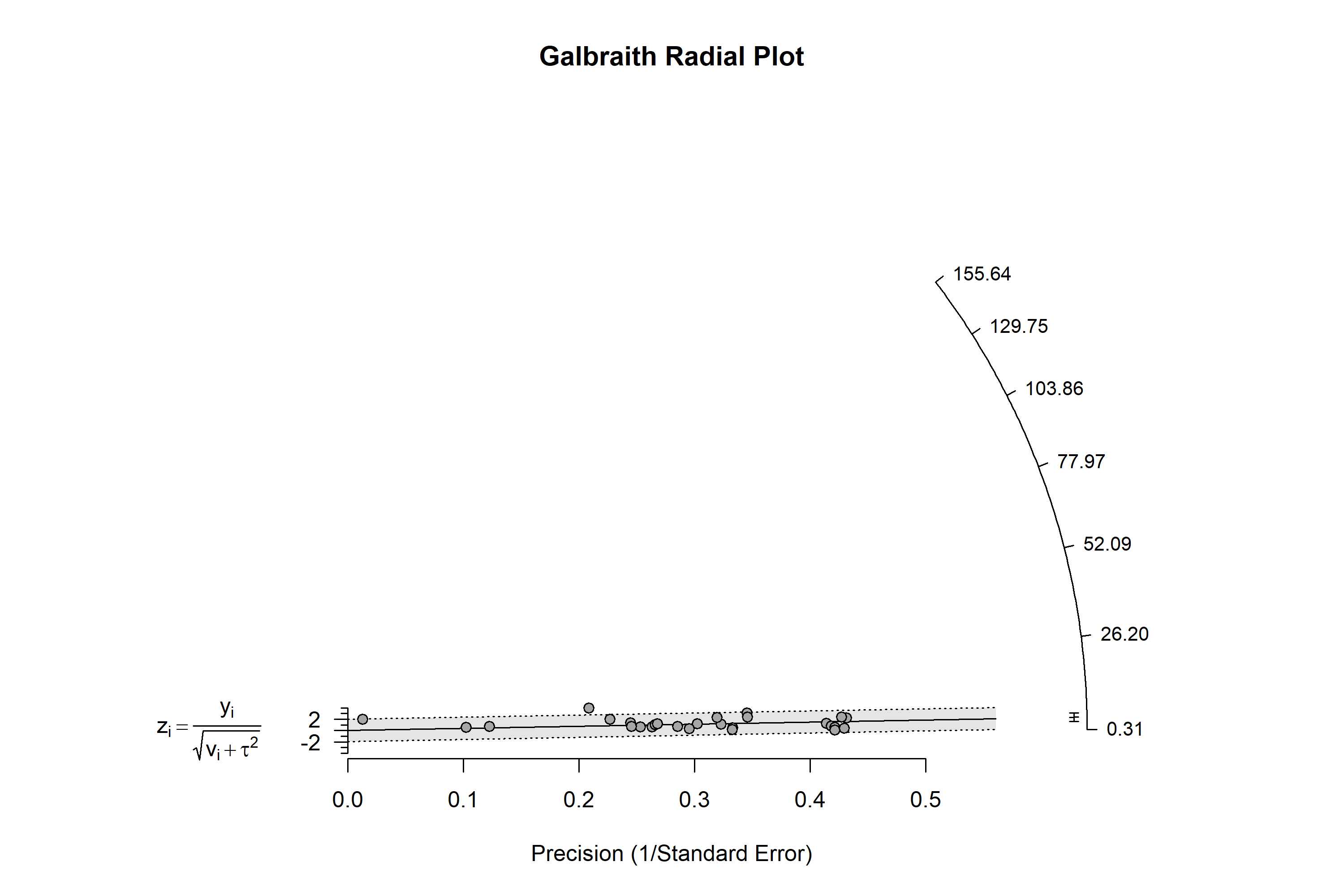

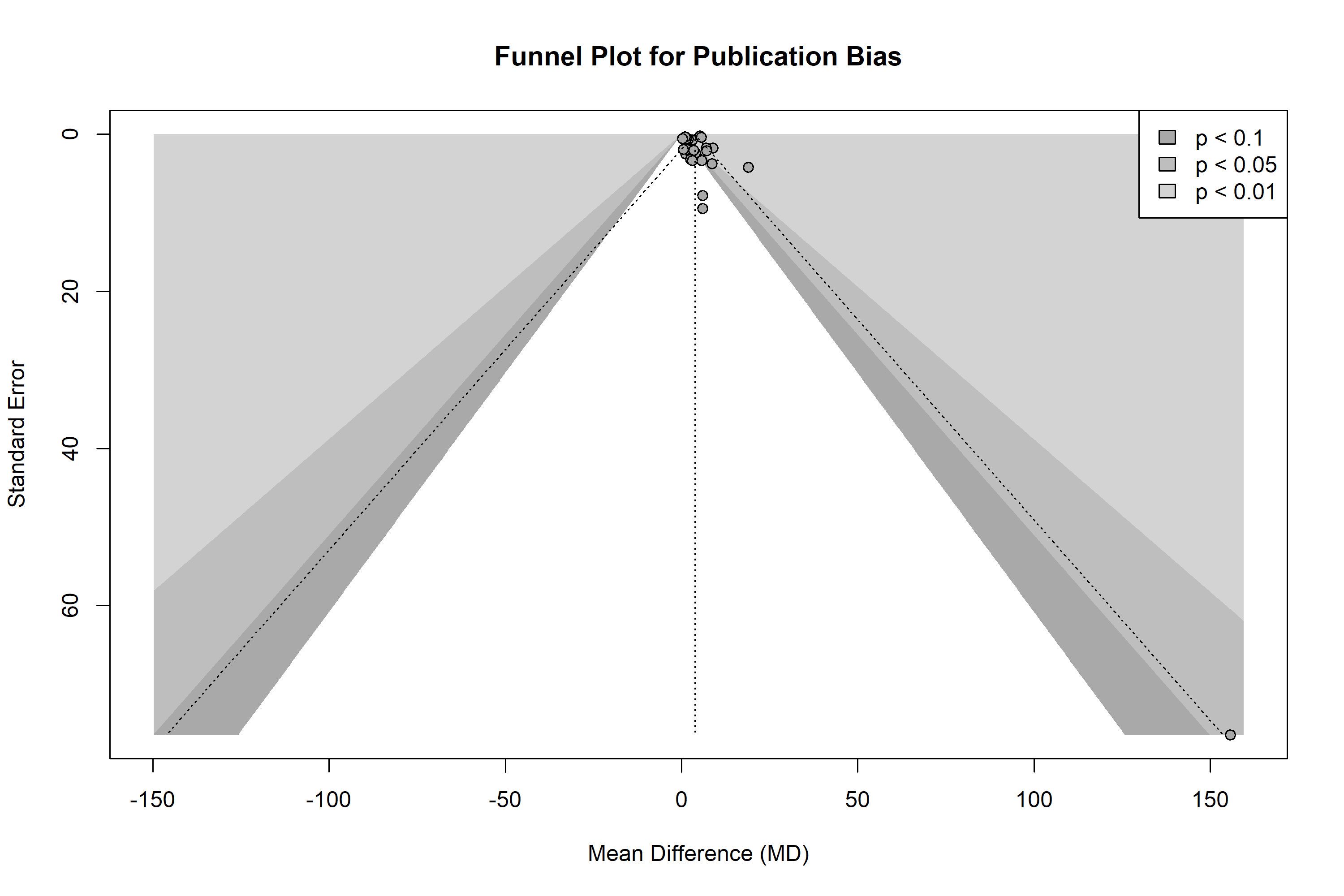


3.6 Outlier Detection (Boxplot)
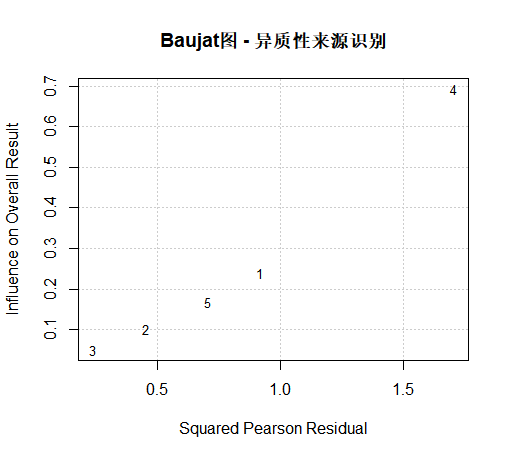

Supplement: Supplementary file 1 [file Supplementaryfile1.doc]
